# Evaluation of Whole Carcass Composting as a Mortality Disposal Option for African Swine Fever Virus-Infected Swine

**DOI:** 10.1155/2023/9926250

**Published:** 2023-06-05

**Authors:** Lindsay Gabbert, Lauren Martignette, Mariceny Zurita, Jose Barrera, John Neilan

**Affiliations:** ^1^U. S. Department of Homeland Security Science and Technology Directorate, Plum Island Animal Disease Center, P.O. Box 848, Greenport, NY 11944, USA; ^2^SAIC, Plum Island Animal Disease Center, P.O. Box 848, Greenport, NY 11944, USA

## Abstract

African swine fever (ASF) epizootic outbreaks often result in the deaths of large numbers of swine either through confirmed or suspected disease or depopulation for outbreak control. Identification of effective biosecure livestock mortality disposal methods, capable of inactivating ASF virus (ASFV) in contaminated carcasses, remains an important component for development of localized outbreak response and recovery plans. In this study, we evaluated ASFV inactivation and viral DNA degradation during composting of ASFV-infected swine carcasses in an Animal Biosafety Level 3-Ag (ABSL3-Ag) high-containment vivarium utilizing virus isolation (VI) and real-time polymerase chain reaction (RT-PCR). Four ASFV-infected pigs, approximately 54 kg, were composted for 37 days in a static, nonaerated windrow assembled using wood chips, horse manure, and pine shavings as the primary sources of carbon. The windrow daily temperature was sustained at ≥55°C for 18 days. Spleen samples, collected over 28 days, were negative for infectious ASFV by virus isolation on day 5 but remained positive for ASFV DNA by RT-PCR through day 28. At the conclusion of the study, bone marrow, muscle, and pinna samples were collected from composted carcasses, in addition to carbon materials in the windrow. No infectious ASFV was detected by virus isolation in any of the decomposed tissues or carbon samples. However, ASFV DNA remained detectable in all swine tissues (*n* = 36) and in 14/16 sentinel carbon samples at the end of the study, indicating that positive detection of ASFV DNA did not correlate with virus infectivity. In conclusion, infectious ASFV was rapidly eliminated in ASFV-infected swine during composting of whole carcasses by day 5, coinciding with a temperature increase in the compost pile to ≥55°C.

## 1. Introduction

African swine fever (ASF) is considered one of the most economically devastating transboundary animal diseases afflicting swine throughout the world. The causative agent, ASF virus (ASFV), is a large nucleocytoplasmic DNA virus and the sole member of the family *Asfarviridae.* Domestic swine (*Sus scrofa domesticus*) infected with highly pathogenic strains of ASFV have high rates of morbidity and mortality resulting in catastrophic numbers of infected swine carcasses during large outbreaks, due to either death or culling of infected animals to curb the spread of disease. Increased outbreaks of ASF in China, Southeast Asia, and more recently in 2021 in the Western Hemisphere (island of Hispaniola) have resulted in the deaths of millions of pigs with economic losses estimated in the hundreds of millions of dollars [1–3].

The lack of licensed vaccines and therapeutics for ASF prophylaxis and treatment, or for use as emergency response tools, has resulted in culling of infected swine as a primary disease control strategy. Implementation of strict biosecurity measures continues to be the most effective way to limit the geographical spread of ASFV among farms.

A critical component of disease control is the biosecure disposal of infected animal carcasses by methods that mitigate further contamination and spread of pathogens [4]. Options for disposal of animal mortalities during large infectious disease outbreaks include deep burial, large-scale shallow burial with carbon, incineration, landfill disposal, rendering, and composting [4–6], with each method having a cost/benefit relationship relative to local resources such as access to carbon material, heavy machinery, labor, and geography.

Previous studies have demonstrated composting of livestock mortality to be an effective method for inactivation of important agricultural viruses. A composting investigation using passively aerated insulated bin systems by Guan et al. [7] reported inactivation of foot-and-mouth-disease virus (FMDV) in foot-and-mouth-disease (FMD) infected pigs within 10 days, and degradation of FMD viral RNA by day 21. Pepin et al. (2021) demonstrated that porcine reproductive and respiratory syndrome virus and porcine epidemic diarrhea virus (PEDV) were noninfectious in swine bioassays after composting preprocessed infected swine carcasses and composting outdoors in cold weather [8]. Results from a Vietnam field study evaluating whole carcass composting of ASFV-infected pigs reported that ASFV was inactivated in spleen tissues after three days in windrows formed using rice hulls [9]. However, no other tissues samples were monitored, including the bone marrow, where ASFV is expected to survive longer within the slowly decomposing and protective bone [10].

In the US, reports on carcass disposal studies utilizing wild-type, virulent ASFV strains are currently lacking due to high biosecurity requirements and stringent laboratory select agent regulations. Thus, research using surrogate viruses, in lieu of wild-type ASFV, have been reported. Ebling et al. (2022) reported that swinepox virus (SwPV), a proposed ASFV surrogate, was noninfectious in spiked swine bone marrow samples subjected to aboveground burial after approximately 11 days [6]. However, it is unknown whether ASFV inactivation directly correlates with that of surrogate DNA viruses, including SwPV, since no head-to-head comparisons have been made.

To better understand the fate of ASFV in swine composted under natural field conditions, we composted ASFV-infected whole swine carcasses in a static windrow-type pile inside a high-containment ABSL-3Ag vivarium. Efforts were made to utilize carbon materials and windrow construction techniques that are currently recommended for use during a large animal disease outbreak in the field. Over 37 days, we evaluated the kinetics of ASFV inactivation in spleen, bone marrow, muscle, and pinna tissue samples and analyzed all samples for the presence of ASFV DNA.

## 2. Materials and Methods

### 2.1. Study Animals

Eight pigs (Yorkshire cross), approximately 54 kg, were provided at the conclusion of an ASFV clinical study conducted by the United States Department of Agriculture (USDA) Agricultural Research Service. The pigs were challenged with wild-type ASFV strain Georgia (Genotype II) via intramuscular inoculation with a 10^2^ 50% hemadsorption dose (HAD) without any additional treatments [11]. After the onset of clinical signs, blood was collected to determine viremia and the pigs were euthanized. Four of the eight pig carcasses (composted pigs 1–4 (CP1–4)) were stored at 4°C for 3 days prior to composting. The four other ASFV-infected pigs (noncomposted Pigs 1–4 (NCP1–4)) were necropsied immediately following euthanasia to obtain spleen, bone marrow, pinna, and muscle tissue samples to determine baseline levels of infectious ASFV. All tissues and blood were stored at −80°C until analysis.

### 2.2. Spleen Sample Preparation

**Figure 1:**
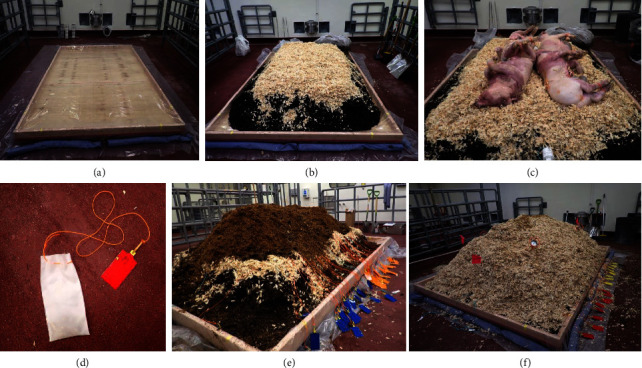
Compost pile construction: (a) wooden platform, poly sheeting, and PIG socks; (b) windrow base consisting of the wood chip and pine shaving layer; (c) swine carcass placement for CP1–4; (d) Dacron sample bags used to place spleen samples back into the swine abdominal cavity; (e) compost pile after addition of spleen samples and core carbon material; (f) final compost pile capped with pine shavings and thermometers placed in each quadrant.

Prior to sample preparation, carcasses used for composting (CP1–4) were removed from cold storage and equilibrated to room temperature overnight. A single incision was made in the abdominal cavity of each pig to extricate the spleen, which was cut into approximately 30 uniform sections. Woven forage bags (10 cm × 20 cm) with a porosity of 50 ± 10 *μ*m (Ankom, R1020) were used to house spleen samples. Each bag was retrofitted with 1.5 m of a high visibility Dacron polyester scuba diving line (SGT Knots (1.85 mm), Amazon.com) and a colored plastic tag for sample identification (Figure 1(d)). One section of spleen was added to each sampling bag in addition to a scoop of the compost windrow core material (horse manure/bedding mix, see below). This was done to better simulate the composting environment external to the bag while allowing microbes and fluids to contact the sample. Twenty-four sampling bags were prepared for each of the four CP pigs. The remaining spleen tissues were frozen at −80°C and used to develop sample processing methods and determine ASFV baseline titers.

### 2.3. Windrow Construction

A windrow-based composting design was adopted in accordance with methods outlined in the USDA Animal Livestock Mortality Composting Protocol [12]. The compost pile was assembled inside a large animal isolation room within the Plum Island Animal Disease Center ABSL3-Ag vivarium. A 2.4 m × 3.0 m wooden base was constructed to contain the carbon material and raise the windrow off the floor. Heavy duty poly sheeting (4 mm) was placed on the top of the platform and surrounding floor. PIG® blue absorbent socks (NewPig, 4048) were placed around the perimeter of the platform to absorb any potential leachate from the windrow (Figure 1(a)). The volume of carbon materials needed for windrow construction was estimated using the swine composting and aboveground burial calculator developed by Flory [13], which calculates the cubic feet of carbon needed for windrow construction based on the total carcass weight. Calculator inputs (four pigs weighing 54 kg each) predicted that a total of 1.8 m^3^ of carbon would be required. The windrow base consisted of approximately 38 cm of municipal woodchips (Southold, NY Department of Solid Waste Management) covered by 10–15 cm of Guardian Premium Pine Flake Shavings (Agway) to act as an adsorbent layer (Figure 1(b)). Carcasses (CP1-4) were arranged in a single layer, back-to-back, with two carcasses per row (Figure 1(c)). Sampling bags (Figure 1(d)) containing infected spleen sections were placed into the abdominal cavity of their respective carcasses, *n* = 24 samples/carcass. The carcasses were covered with a prewetted core mixture comprising a 1 : 3 mix of horse manure and stable bedding (Southold, NY Department of Solid Waste Management) (Figure 1(e)). The compost pile was capped with 30–38 cm of Guardian Premium Pine Flake Shavings (Agway) (Figure 1(f)).

### 2.4. Temperature Monitoring

Three methods were utilized to measure compost pile temperatures over the duration of the study. Stainless steel compost thermometers (91 cm long, 0.8 cm diameter) outfitted with probe handles and guards (Model A36PF, ReoTemp) were left inserted in each quadrant of the pile and observed daily to obtain manual temperature readings at a depth of 46 cm. A HOBO U-12 data logger (Model U12-015-02, Onset, Bourne, MA), set to record temperatures every 10 minutes for infinity, was inserted into the rectum of CP2 to measure temperatures in the carcass throughout the composting experiment. Additionally, a HOBO 4-channel thermocouple data logger (Model UX120-014M, Onset, Bourne, MA) outfitted with four 12 m Type-J thermocouple wires (Part TCW100-J) was installed in the viewing window of the animal isolation room to enable monitoring of windrow temperatures from the exterior of the room. One thermocouple wire recorded ambient room temperature, while the remaining three thermocouples were inserted at different depths in the compost pile. Thermocouple temperatures were recorded twice daily by second- and third-shift personnel.

### 2.5. Sample Collection

#### 2.5.1. Spleen + Compost

To minimize disruption of the carcasses and windrow during composting, spleen tissues were the only clinical samples monitored longitudinally and were collected on study days 0, 1, 3, 5, 7, 10, 14, 21, and 28. Dacron sample bags containing a piece of the ASFV-infected spleen and compost core material were manually removed from each of CP1–4 in triplicate at each sampling timepoint. This was easily accomplished by pulling the sample tag attached to the Dacron line connected to each sample bag. The sample bags were immediately placed into individual plastic bags and sealed to prevent cross-contamination. The contents of each bag were aseptically transferred to a 50 mL conical tube in a class II biosafety cabinet and stored frozen at −80°C.

#### 2.5.2. Swine Tissues

After 37 days, the windrow was disassembled and final tissue samples were collected. The carcasses were in advanced states of decomposition, with soft tissues mostly unidentifiable. Tissues available for collection included bone marrow from the femur, muscle from the hind quarter, and pinna (skin/cartilage from the outer ear). Three replicates of each tissue type were collected from CP1–4 and stored at −80°C until homogenization. Similar tissues were collected from NCP1–4 immediately after euthanasia to determine baseline levels of infectious ASFV and ASFV DNA (Section 2.1).

#### 2.5.3. Carbon Materials

Sixteen grab samples of carbon material, chosen at random from the deconstructed windrow, were collected in sterile 50 mL conical tubes. The samples were representative of each of the three carbon materials used in construction of the pile (wood chips, pine shavings, and the core horse manure/bedding mix) and were stored at −80°C.

### 2.6. Sample Processing

#### 2.6.1. Homogenization of Spleen Tissues and Carbon Materials

To facilitate homogenization, 2 g of sterile sand (Fisher, S25-3) was added to each sample of spleen and compost material after thawing on ice. The samples were manually macerated with a mortar and pestle until they reached a paste-like consistency. A 1.5 ml storage tube was loosely filled with this material and frozen at −80°C until extraction of viral DNA and analysis via RT-PCR (Section 2.7).

The remainder of the sample homogenate was resuspended with 25 ml of Roswell Park Memorial Institute (RPMI) 1640 Media (Gibco, 21870-084) containing 3% antibiotic-antimycotic (A/A) (Gibco, 15240-062) by direct addition to the mortar. Sample extracts were decanted into 50 ml conical tubes on ice and centrifuged at 3,000 × g for 10 minutes to remove large solids. The supernatant was passed through a series of 0.45 *μ*m syringe filters (Whatman, 6896-2504) and stored in aliquots at −80°C until virus isolation (Section 2.8). The samples of carbon materials collected from the deconstructed compost pile on day 37 were also processed as described above.

#### 2.6.2. Homogenization of Bone Marrow, Muscle, and Pinna Tissues Collected on Days 0 and 37

Tissue samples were collected in quadruplicate from NCP1–4 at day 0 and CP1–4 at day 37 and homogenized using a TissueLyser II (Qiagen, 85300). Briefly, a 1 cm^3^ piece of tissue was added to a 2 mL cryovial containing a 5 mm stainless steel bead (Qiagen, 69989) and 1 mL RPMI media +2% A/A and homogenized for 2 minutes at 30 Hz. The samples were centrifuged at 10,600 × g for 2 minutes, and the supernatants were used for virus isolation and quantification and to extract DNA for RT-PCR analysis.

### 2.7. DNA Extraction and RT-PCR

Total DNA was extracted from tissue supernatants (Sections 2.6.1 and 2.11) and whole blood using the DNeasy Blood & Tissue Kit (Qiagen, 69506). DNA from compost homogenates obtained from sectioned spleen samples and carbon materials (Section 2.6.2) was extracted using the DNeasy PowerSoil Pro kit (Qiagen, 47016). Briefly, 0.25 g of the homogenate obtained after manual maceration was transferred to a power bead tube included with the kit. Two *μ*L of VetMAX™ Xeno™ Internal Positive Control (IPC) DNA (10,000 copies/*μ*L) was added to each sample to monitor the presence of PCR inhibitors during DNA purification before proceeding according to the manufacturer instructions.

Extracted DNA was amplified on the Applied Biosystems 7500 instrument using a one-step RT-PCR assay targeting the highly conserved ASFV p72 structural protein as previously described [14]. Cycling conditions consisted of a denaturation step at 95°C for 20 minutes, followed by 45 cycles of a two step: 10 seconds at 95°C and 30 seconds at 60°C. Samples containing Xeno™ IPC were multiplexed using the VetMAX™ Xeno™ Internal Positive Control-LIZ™ assay (ThermoFisher, A29766) using the primers and probe (CY5 reporter) provided in the kit. Threshold cycle (Ct) values less than 40 were considered positive for ASFV DNA.

### 2.8. Virus Isolation and Titration

#### 2.8.1. Monocyte-Derived Macrophage Harvest and Preparation

Monocyte-derived macrophage (MDM) cultures were prepared as previously described for use in ASFV isolation and quantification [15]. Briefly, EDTA-treated whole blood, obtained from healthy donor pigs, was incubated in a 37°C water bath for one hour to separate erythrocytes from white blood cells. Monocytes were further separated by flotation over Ficoll Paque (Cytiva, 17544202) and density gradient centrifugation. MDMs were washed three times before overnight incubation in T75 Primaria flasks (Corning, 353810) in base media (RPMI 1640 medium (Gibco, 21870-084)) with a 30% L929 supernatant [16], 20% fetal bovine serum (FBS; SAFC, 12103C), and 30% sterile-filtered blood plasma. Adherent MDMs were harvested from flasks by detachment in phosphate-buffered saline (DPBS) (Gibco, 14190-144) containing 2% 0.5 M EDTA (Gibco, 15575-020) and washed prior to seeding directly in 6- or 96-well Primaria plates (Corning, 353846 or 353872).

#### 2.8.2. Virus Isolation Assay for Infectious ASFV

MDMs were seeded at 2.0 × 10^6^ cells/well in 6-well Primaria plates (Corning, 353846) and incubated overnight in base media. Clarified supernatants from tissue and compost samples were thawed, serially diluted in base media (10^0^−10^−2^), and plated into wells containing a 25% solution of washed swine red blood cells (RBCs) in DPBS (2% RBC in the total volume of media per well). The plates were incubated for 7 days at 37°C (5% CO_2_) and scored for the presence of hemadsorption (HAD) based on visual observation of rosette formation. Negative samples were passaged a total of three times to confirm the absence of infectious ASFV.

#### 2.8.3. Determination of Viral Titers

Samples positive by the HAD assay were titrated to quantify infectious ASFV levels. MDMs (1.25 × 10^4^ cells/well) were seeded in 96-well Primaria plates (Corning, 353872) in base media and incubated overnight. Sample supernatants were serially diluted in RPMI base media containing swine red blood cells (RBCs) diluted 25% in sterile DPBS. 100 *μ*L of each dilution was added to wells in replicates of 8, and the plates were incubated for 7 days at 37°C in 5% CO_2_. Cells were observed for the presence of HAD and scored based on formation of rosettes. Titers were calculated using the Reed–Muench method [17] and expressed as log_10_ 50% hemadsorption dose (HAD_50_)/mL. Similarly, ASFV was quantified in blood and tissue samples collected on day 0.

### 2.9. In Vitro Tissue Heat Treatment

One-centimeter pieces of the bone marrow, pinna, and muscle, harvested from ASFV-infected swine (NCP 1–4), were transferred to individual microcentrifuge tubes and placed inside an environmental chamber (BINDER model KMF-240) set at 55°C. The tissues were incubated for both 24 and 72 hours, homogenized using the TissueLyser II protocol described above (Section 2.6.2), and analyzed for the presence of infectious ASFV by VI (Section 2.8.2). Additionally, ASFV DNA was extracted as described above (Section 2.7) to measure degradation after heat treatment by RT-PCR.

## 3. Results

### 3.1. Compost Windrow Temperatures

**Figure 2:**
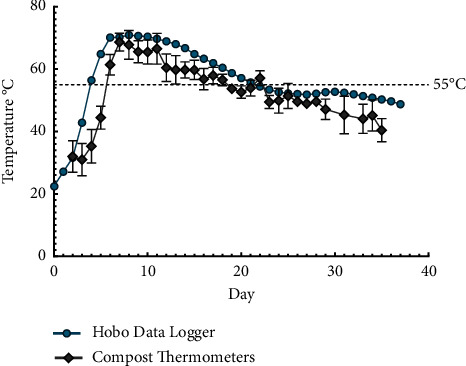
Compost pile temperatures: manual compost thermometer vs. HOBO data logger. The daily means from the manual compost thermometers were the average of four readings taken from each quadrant of the compost pile. The HOBO data logger recorded the rectal temperature of composted pig number 2.

The mean ambient air temperature in the animal isolation room was 21.5°C throughout the experiment duration with an average relative humidity of 41.8%. The compost pile reached the thermophilic or high-temperature phase of 55°C by day 5 and maintained elevated temperatures ≥55°C for 18 days. Peak temperatures of 70–76°C occurred on days 6–13 (Figure 2). At the study conclusion (day 37), a temperature of 48.8°C was recorded. Temperature measurements obtained from the buried HOBO U-12 data logger were consistent with the daily reads obtained manually with the compost thermometers (Figure 2), albeit trending slightly higher throughout. The Type-J thermocouple wires attached to the HOBO 4-channel thermocouple data logger became corroded after contact with organic matter in the pile and failed by day 9 (data not shown).

### 3.2. Infectious ASFV Tissue Measurements

**Figure 3:**
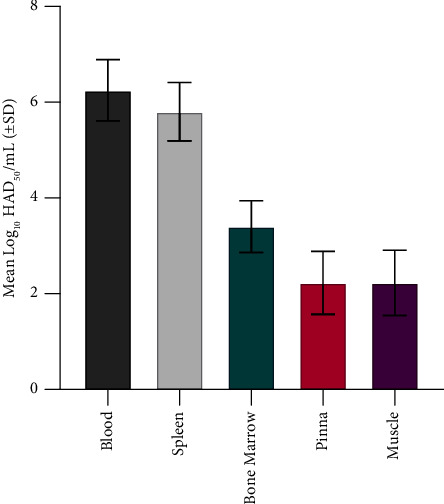
Baseline titers of infectious ASFV in noncomposted pig blood and tissues. Four samples from each tissue were analyzed. HAD_50_, 50% hemadsorption dose; SD, standard deviation.

Infectious ASFV baseline titers were determined for noncomposted swine tissues including blood, spleen, bone marrow, muscle, and pinna (Figure 3). HAD_50_ values were the highest in whole blood and spleen with average titers of 6.3 and 5.8 log_10_, respectively. Bone marrow samples had higher levels of ASFV than pinna or muscle, with an average HAD_50_ titer of 3.4 log_10_ compared with 2.2 log_10_ in both pinna and muscle (Figure 3).

All spleen samples collected from composting pigs were positive for ASFV by VI on days 0 and 1 (Table 1). On day 3, only 1 of 12 samples was positive after further amplification by a second passage in tissue culture, suggesting significant inactivation occurred by that timepoint. Three replicate spleen samples collected from each of CP 1–4 on study days 5, 7, and 10 were passaged three times on MDMs and were HAD negative, confirming that ASFV inactivation in spleen samples from actively composting carcasses was complete by day 5. Therefore, samples from the remaining study timepoints (14, 21, and 28 days) were not analyzed.

Bone marrow, muscle, and pinna samples, collected from each carcass (CP1–4) on day 37, were processed in triplicate (*n* = 12 per tissue type), and all samples were negative after 3 passages on MDMs as well (data not shown).

### 3.3. Detection of ASFV DNA by RT-PCR

**Figure 4:**
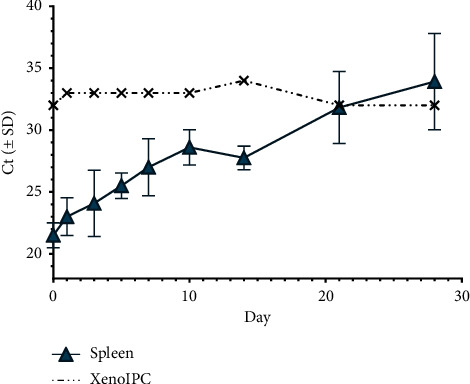
RT-PCR results for spleen samples collected from composted pigs over 28 days. Triplicate samples/timepoint. Xeno IPC was an internal DNA control.

RT-PCR results for detection of ASFV DNA in composted spleen tissues demonstrated degradation over time, with average cycle threshold (Ct) values of 21.5 on day 0 increasing to 33.9 by day 28 (Figure 4). Despite the complex biological nature of the samples and high levels of organic and/or decaying tissue, inhibition of the RT-PCR reaction was not observed. This was supported by the stable recovery and detection (average Ct value of 32.8) of Xeno™ Internal Positive Control DNA after addition to samples from all collection timepoints (Figure 4).

**Figure 5:**
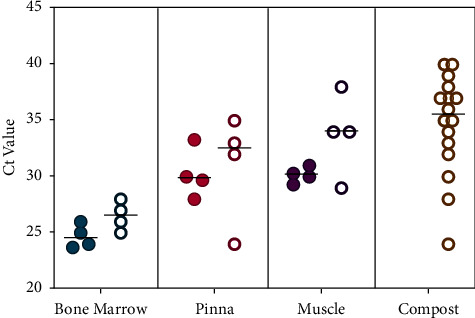
Detection of ASFV DNA via RT-PCR in noncomposted swine tissues at day 0 (open symbols) and in composted swine tissues and carbon materials after 37 days (closed symbols). Data points for tissues represent the average Ct value of three replicate samples tested by RT-PCR from each pig (CP1–4).

All tissue samples collected on day 37 (bone marrow, muscle, and pinna) were positive for ASFV DNA by RT-PCR, with the highest levels of DNA detected in the bone marrow (average Ct 26.5), followed by in the pinna (average Ct 31.0) and muscle (average Ct 33.8). Among the three tissue types, comparing those composted for 37 days vs. noncomposted fresh tissues (Figure 5), the average differences in Ct values were 1.8, 0.7, and 3.8 for the bone marrow, pinna, and muscle, respectively, suggesting that heat, microbial activity, and biological decomposition had minor effects on ASFV DNA stability and recovery. Similarly, in compost carbon materials collected on day 37, ASFV DNA was detected in 14/16 samples. However, RT-PCR results exhibited a wide range of Ct values from 24–40 (Figure 5).

### 3.4. In Vitro Heat Treatment of ASFV-Infected Tissues

**Figure 6:**
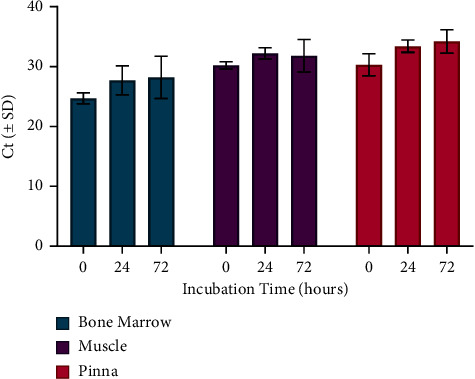
Effects of incubation at 55°C on the detection of ASFV DNA by RT-PCR in noncomposted swine tissues (NCP1-4). Each point represents the average Ct value and standard deviation (SD) for the 4 pigs.

All tissues collected from each of NCP1–4 (bone marrow, muscle, and pinna) incubated at 55°C for 24 hours were negative for infectious ASFV by VI after three blind passages on MDMs (data not shown). ASFV DNA was detectable in all heat-treated tissues at both the 24 and 72 hr timepoints (Figure 6).

## 4. Discussion

During a transboundary animal disease outbreak, the goals of carcass disposal include (1) maintaining on-farm biosecurity of infected premises to minimize the spread of a virus to the surrounding environment, (2) mitigating detrimental short- and long-term environmental impacts, and (3) pathogen inactivation in contaminated carcasses. Thus, in locations with adequate access to carbon resources, composting has been identified as a cost-effective disposal option for emergency management of animal mortalities [5], due to the fact that it can be implemented directly on farms, limits the need to transport infected carcasses offsite, and results in timely inactivation of viruses [4, 5, 18, 19].

Composting results in pathogen inactivation through exposure to high heat and microbial action over time. By conducting this study in a controlled laboratory environment, we were able to directly monitor the effects of composting on ASFV inactivation while minimizing the potential impact of environmental variables such as weather, insects, and scavengers. We measured inactivation of >5 log_10_ ASFV in spleen tissues by day 5 of composting, coinciding with the initial increase in the temperature in the compost pile. These results are consistent with other published data supporting ASFV inactivation in the spleen by day 3 in composted ASFV-infected pigs [9].

Detection of ASFV DNA in the majority of all analyzed samples was expected due to the known stability of the double-stranded ASFV DNA genome and the small size of the PCR amplicon. For example, ASFV p72 DNA was detected in the bone marrow of excavated wild boar skeletons after burial for two years [20]. A study comparing survival of human adenovirus 41 (Ad41), a DNA virus, with two RNA enteric viruses (murine norovirus and Aichi virus) demonstrated that Ad41 was rendered noninfectious after one day during composting of biosolids. However, its genome was more stable than that of both RNA viruses [21]. In contrast, composting studies utilizing RNA viruses of agricultural significance such as highly pathogenic avian influenza (HPAI), Newcastle disease virus (NDV), FMDV, and PEDV demonstrated that viral RNA is degraded to nondetectable levels by day 10 (HPAI and NDV) [22], day 21 (FMDV) [7], or day 36 (PEDV) [23].

The high frequency of positive ASFV DNA samples in the current study demonstrated that RT-PCR was not an appropriate method to confirm the absence of infectious ASFV in composted carcasses or carbon materials. Consideration of other pathogen detection methods will be important for development of ASF outbreak recovery and swine restocking protocols, including environmental sampling. Current U.S. Environmental Protection Agency standards for release of class A compost require maintaining temperatures between 55°C and 70°C for three consecutive days for static aerated piles [24]. This policy has also been adopted into the USDA Mortality Composting protocols for diseases such as HPAI [25, 26]. Our results support generation of similar policies for ASF, due to the loss of ASFV infectivity at early timepoints during composting.

We determined through *in vitro* experiments that heat alone inactivated high titers of ASFV in three tissues after incubation at 55°C for 24 hours. Therefore, exposure to temperatures typically reached during composting may be sufficient to rapidly eliminate infectious ASFV without a requirement for additional microbial activity or biological decomposition. The diagnostic standard for *in vitro* detection of infectious ASFV requires 3 blind sample passages in tissue culture to confirm negativity. Although this standard was followed in our study, other composting research utilized bioassays in animals to further confirm virus inactivation. For example, in composted piglets, infectious pseudorabies virus (PRV), an enveloped, double-stranded DNA virus, was not detectable via tissue culture in compost samples by day 7, and when the same composted carbon materials were blended with feed and fed continuously to naïve swine, no clinical signs of PRV were observed [27].

The current study utilized a static compost pile design and was intentionally not turned to encourage a second round of microbial decomposition. This allowed for verification that ASFV inactivation occurred during the early stage of composting and also reduced laboratory biosafety risks. Turning of windrows in the field after the initial high-temperature (thermophilic) composting phase is often performed to encourage a greater degree, or a faster rate, of carcass decomposition, especially for larger livestock. Enhanced decomposition can also be accomplished by preprocessing of carcasses by grinding, which potentially allows for use of smaller volumes of carbon material, less land space for windrows, and accelerated tissue decomposition compared to more traditional whole carcass methods [8]. The experimental results presented support inactivation of ASFV at early timepoints in successfully constructed compost piles; thus, turning of piles after maintaining the desired temperature should not present further biosecurity risks by spreading infectious ASFV in the environment.

## 5. Conclusions

In conclusion, no infectious ASFV was detected in any swine tissue tested after composting ASFV-infected whole carcasses for 37 days. Longitudinal sampling revealed that initially high titers of ASFV in the spleen were nondetectable by day 5, indicating virus elimination in composted carcass tissues at early timepoints after windrow temperatures became elevated. Therefore, if adequate resources are available and environmental conditions are favorable during an ASFV outbreak, windrow-based composting shows promise as an effective means of mortality disposal for ASFV-infected swine.

## Figures and Tables

**Table 1:** Results of virus isolation testing for spleen samples (*n* = 3) collected from CP1-4 on study days 0, 1, 3, 5, 7, and 10.

Day of collection																			
	Sample replicate	0			1			3			5			7			10		
		Pass 1	Pass 2	Pass 3	Pass 1	Pass 2	Pass 3	Pass 1	Pass 2	Pass 3	Pass 1	Pass 2	Pass 3	Pass 1	Pass 2	Pass 3	Pass 1	Pass 2	Pass 3
CP1	A	+	nt	nt	+	nt	nt	—	—	—	—	—	—	—	—	—	—	—	—
	B	+	nt	nt	+	nt	nt	—	—	—	—	—	—	—	—	—	—	—	—
	C	+	nt	nt	+	nt	nt	—	—	—	—	—	—	—	—	—	—	—	—

CP2	A	+	nt	nt	+	nt	nt	—	—	—	—	—	—	—	—	—	—	—	—
	B	+	nt	nt	+	nt	nt	—	—	—	—	—	—	—	—	—	—	—	—
	C	+	nt	Nt	+	nt	nt	—	—	—	—	—	—	—	—	—	—	—	—

CP3	A	+	nt	nt	+	nt	nt	—	—	—	—	—	—	—	—	—	—	—	—
	B	+	nt	nt	+	nt	nt	—	—	—	—	—	—	—	—	—	—	—	—
	C	+	nt	nt	+	nt	nt	—	—	—	—	—	—	—	—	—	—	—	—

CP4	A	+	nt	nt	+	nt	nt	—	—	—	—	—	—	—	—	—	—	—	—
	B	+	nt	nt	+	nt	nt	—	—	—	—	—	—	—	—	—	—	—	—
	C	+	nt	nt	+	nt	nt	—	+	+	—	—	—	—	—	—	—	—	—

(+/−): positive/negative for hemadsorption; nt = not tested.

## Data Availability

The primary data used to support the findings of this study are included within the article.
